# Effects of mental fatigue on agility and coordination in female soccer players under high ambient temperature

**DOI:** 10.3389/fphys.2026.1964642

**Published:** 2026-09-21

**Authors:** Yuankang Wang, Yimei Duan, Guotian Lu, Mengda Wu

**Affiliations:** School of Physical Education, Sichuan Normal University, Chengdu, China

**Keywords:** agility, coordination, high ambient temperature, mental fatigue, soccer players

## Abstract

**Objective:**

To explore the effects of mental fatigue (MF) induced under high ambient temperature on agility and coordination in female soccer players.

**Methods:**

A randomized crossover design was adopted. Twenty-four female soccer players were recruited, and 19 participants completed all experimental sessions in a randomized balanced crossover design under high temperature (≥32 °C) or normal temperature (23 ± 1 °C), including either a 45-min Stroop task or a neutral video-watching control task followed by the Illinois agility test and the Harre circuit test, with a one-week washout period. All 19 participants were included in the final statistical analyses.Two-way repeated-measures ANOVA was used to examine the main and interactive effects of ambient temperature and MF.

**Results:**

The 45-min Stroop task successfully induced a mental fatigue state. High ambient temperature was associated with an overall elevation in subjective mental fatigue levels, with a large main effect size (*p* < 0.001, η²p=0.713). For agility, both MF and high temperature exerted independent detrimental main effects: MF significantly prolonged agility performance time (*p* < 0.001, η²p=0.603), and high temperature also significantly impaired agility (*p* = 0.002, η²p=0.414), with no significant interaction (*p* = 0.102). For coordination, a significant interaction was found between temperature and MF (*p* < 0.001). Simple effects analysis showed that MF had no significant effect on coordination under normal temperature (p = 0.751), and high temperature alone did not impair coordination (p = 0.757). However, the combination of high temperature and MF significantly deteriorated coordination (*p* < 0.001, d_z_ = 1.247, 95% CI = 0.604~1.889). In addition, both MF and high temperature independently increased ratings of perceived exertion (RPE, *p ≤* 0.001), while the decline in motivation was mainly attributed to high temperature (*p* = 0.003, η²p=0.397).

**Conclusion:**

No significant interaction was detected between ambient temperature and mental fatigue for agility performance, and the results were compatible with an independent additive pattern. For coordination, a significant Temperature × MF interaction effect was observed, whereby coordination performance was only significantly impaired when high ambient temperature and mental fatigue co-occurred. It is recommended to strictly manage athletes’ cognitive load before matches in hot environments to reduce the risk of MF and maintain stable performance of specific physical qualities.

## 1 Introduction

The outcome of soccer matches often depends on the players’ ability to efficiently execute technical and tactical actions in complex and ever-changing pitch environments, among which agility and coordination serve as the fundamental bases for performing breakthroughs, interceptions, and movement transitions (Morgans et al., 2014). In elite-level soccer competition, any subtle internal or external factors may influence the match outcome (Modric et al., 2023), Among external environmental factors, high ambient temperature is a common and severe challenge in tournaments held during summer or in tropical region (Draper et al., 2023; Kang et al., 2024). Studies have shown that high ambient temperature induces physiological stress, including thermoregulatory imbalance and increased cardiovascular strain, thereby accelerating fatigue, prompting premature adjustments in exercise intensity, limiting athletic capacity, and consequently impairing both aerobic and anaerobic performance (Adams et al., 2016; Douzi et al., 2020; Liu et al., 2022; Nassis et al., 2024), Among internal physiological factors, MF is a psychophysiological negative state resulting from prolonged cognitive task performance, characterized by the depletion of central nervous system (e.g., prefrontal cortex) resources, along with decreased motivation, increased perception of effort, and slowed reaction speed (Marcora et al., 2009). Meanwhile, soccer is a sport with high cognitive demands, requiring players to continuously engage in rapid decision-making, attention allocation, and response inhibition during training and competition, which predisposes them to a state of mental fatigue (MF) (Smith et al., 2018; Thompson et al., 2020). Studies conducted under normal-temperature conditions have shown that this MF state not only negatively affects physical performance—impairing coordination and aerobic capacity, but not agility—but also compromises technical, tactical, and decision-making performance (Martin et al., 2018; Gantois et al., 2020; Batista et al., 2021).Furthermore, most existing research on the effects of MF on athletic performance has been carried out under normal-temperature conditions, leaving the impact of MF under high ambient temperature—a common real-world training and competition environment—insufficiently explored. Considering the actual circumstances of competitive matches, the physiological stress induced by high temperature and the cognitive load inherent to match play often co-exist, and their combination may exert complex interactive effects on players’ physical performance. On this basis, the present study proposed the following hypotheses:

  Hypothesis 1: Both high ambient temperature and MF will exert independent negative main effects on agility in female soccer players, with no significant interaction between the two factors (i.e., the detrimental effects are additive).
  Hypothesis 2: High ambient temperature and MF will produce a significant negative synergistic interactive effect on coordination in female soccer players.

## 2 Participants and methods

### 2.1 Participants

G*Power 3.1 software (Franz Faul, Universität Kiel, Germany) was used to estimate the required sample size *a priori*. The full parameters for the repeated-measures ANOVA (within factors) were set as follows: test family = F tests, effect size f=0.33, α error probability = 0.05, power (1−β error probability) = 0.80, number of groups = 1 (crossover design, all participants received all conditions), number of measurements = 4 (2 ambient temperature × 2 MF conditions), correlation among repeated measures = 0.5, nonsphericity correctionϵ=1. The effect size for sample size estimation in this study was primarily derived from the findings of Smith et al. (2015), which reported that the effect sizes (f) for the impact of MF on technical and cognitive performance in soccer players ranged from 0.48 to 0.76, the median value of 0.66 was used for estimation. To accommodate the present experimental design (Repeated-Measures ANOVA), Cohen’s d (corresponding to a mean of 0.66) was converted to Cohen’s f suitable for the repeated-measures model, yielding an estimated effect size of f ≈ 0.33.

The minimum required sample size was estimated to be 16 participants. Accounting for a potential dropout rate of 20%, a total of 24 female collegiate soccer players with a high competitive level were ultimately recruited to participate in the study, According to the McKay et al. (2021) Participant Classification Framework, participants were classified as Tier 3: Highly Trained/National Level athletes. All athletes held national second-tier athlete certification or above, with an average of 6.2 ± 2.1 years of systematic soccer training experience. They trained 4–5 times per week with a total weekly training volume of 8–10 hours, including technical, physical, and tactical sessions, and competed regularly in the National College Soccer League. No formal heat acclimatization training was conducted prior to the experiment. Participants had a mean age of 20.06 ± 3.54 years, height of 164.50 ± 5.66 cm, and body mass of 55.9 ± 1.41 kg. During the experimental period, five participants dropped out due to personal reasons or injuries, resulting in 19 participants completing all experimental sessions.

The inclusion criteria were as follows: (1) no history of major surgery or acute injury within the three months prior to the experiment; (2) not taking medications that could affect athletic performance (e.g., β-blockers); (3) no communication or cognitive impairments; and (4) no participation in other interventional studies within the preceding three months. To control for response bias, the true purpose and core hypotheses of the experiment were concealed from participants throughout the study. Participants were informed that the investigation aimed to “examine the effects of pre-training cognitive activities on perceptual and physiological responses during agility and coordination training in soccer players under high-temperature conditions.” No participant deduced the core research purpose during the experiment. This study was approved by the Ethics Committee of Sichuan Normal University (Approval No.: 2026LS0139) and was conducted in accordance with the 1964 Declaration of Helsinki and its later amendments. All participants were informed of the experimental procedures and potential risks, and provided written informed consent prior to participation.

### 2.2 Study design and procedures

This study employed a randomized crossover design with a 2 × 2 factorial design for blocking. The independent variables were ambient temperature (high vs. normal) and MF induction (yes vs. no), resulting in four experimental conditions: high-temperature MF condition (HF), high-temperature control condition (HC), normal-temperature MF condition (NF), and normal-temperature control condition (NC). Each intervention session was separated by an interval of at least 7 days (washout period) to ensure complete elimination of residual effects from heat exposure and fatigue. A Latin square design was adopted to counterbalance the order of treatments, thereby offsetting order effects and minimizing learning effects (**Figure 1**).

**Figure 1:**
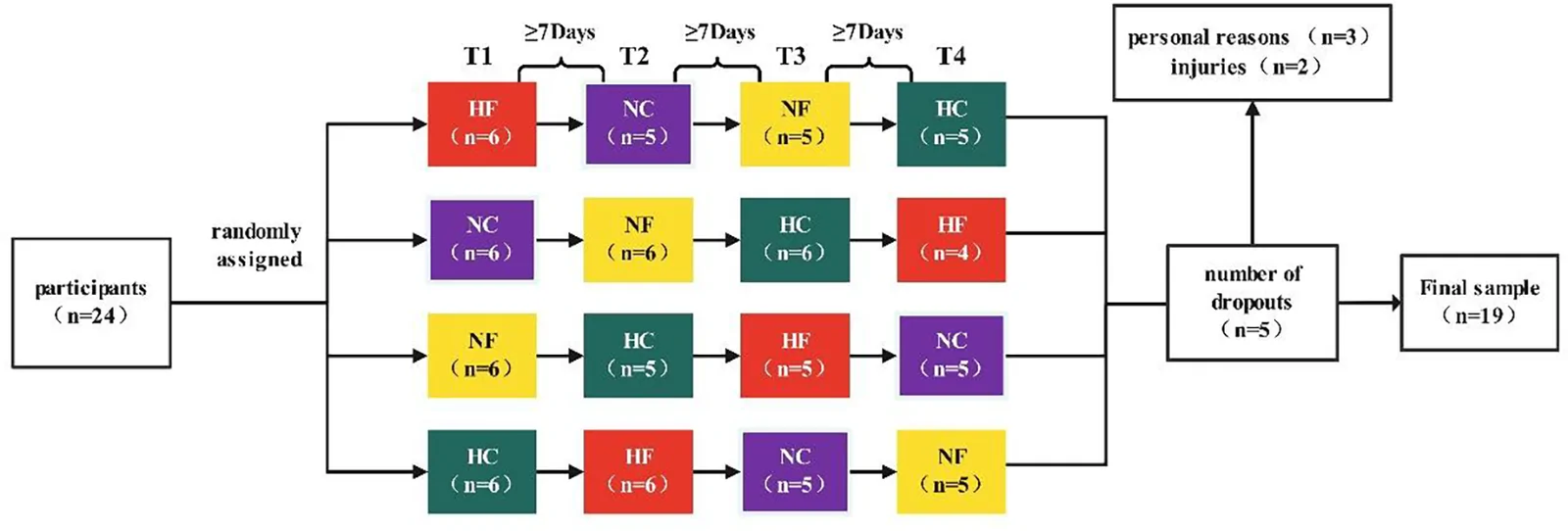
Intervention order diagram. HF, high-temperature MF; HC, high-temperature control; NF, normal-temperature MF; NC, normal-temperature control. T1–T4 denote the four testing sessions in chronological order.

Participants visited the laboratory on a total of five occasions, with the first session serving as a familiarization session and the remaining four as formal experimental sessions. To ensure scientific rigor and smooth implementation, the following content was delivered during the first familiarization session: (1) introduction to the Visual Analog Scale (VAS) for rating mental fatigue and motivation, and the Ratings of Perceived Exertion (RPE) scale, along with their instructions for use; (2) familiarization and brief hands-on practice with the Stroop task and the multiple reaction time apparatus (Model BD-II-509B); (3) familiarization with the movement routes, start and stop criteria for the agility test (Illinois Agility Test) and the coordination test (Harre Circuit Test); and (4) instructions for participants to maintain adequate sleep during the experimental period, refrain from vigorous exercise and from ingesting products containing substances that may enhance athletic performance or cognitive function (e.g., caffeine, L-theanine) within 24 hours prior to each session, and avoid prolonged or high-intensity cognitive activities (e.g., playing computer or mobile games) on the day of testing.

The four formal experimental sessions followed identical procedures. Participants were randomly assigned to four groups, and each group completed all four intervention combinations (i.e., HF, HC, NF, and NC) in a randomized, counterbalanced order. The specific procedures for each of the four sessions were as follows (**Figure 2**): before the intervention, participants’ Choice Reaction Time (CRT), VAS-MF score, and VAS-MO (motivation) score were measured. The HF and NF groups then underwent a 45-minute Stroop task intervention, while the HC and NC groups watched a 45-minute neutral video as a control treatment (the HF and HC groups received their interventions under high-temperature conditions, whereas the NF and NC groups did so under normal-temperature conditions). Immediately after the intervention, VAS-MF, VAS-MO, and CRT were reassessed. Following a standardized 5-minute warm-up consisting of light jogging, dynamic stretching and brief change-of-direction drills, participants completed the agility test (Illinois Agility Test) first and then the coordination test (Harre Circuit Test) in a fixed order, with each test performed twice, separated by a 2-minute rest interval, and a 2-minute rest interval between the two tests. Ratings of Perceived Exertion (RPE) were collected immediately after each test, and VAS-MF and CRT were measured again after both tests were completed. To minimize assessment bias, a partial blinding strategy was implemented. Given the distinct nature of the cognitive tasks (Stroop task vs. neutral documentary) and the perceptible difference in ambient temperature conditions, participants were not blinded to the specific intervention conditions. However, the true purpose and core hypotheses of the study remained concealed from participants throughout the experiment. In addition, the outcome assessors responsible for timing the agility and coordination tests were blinded to each participant’s intervention allocation. The MF induction procedures and physical performance tests were conducted by separate teams of trained researchers to preserve assessor blinding. No assessor unblinding occurred during the experimental period.

**Figure 2:**
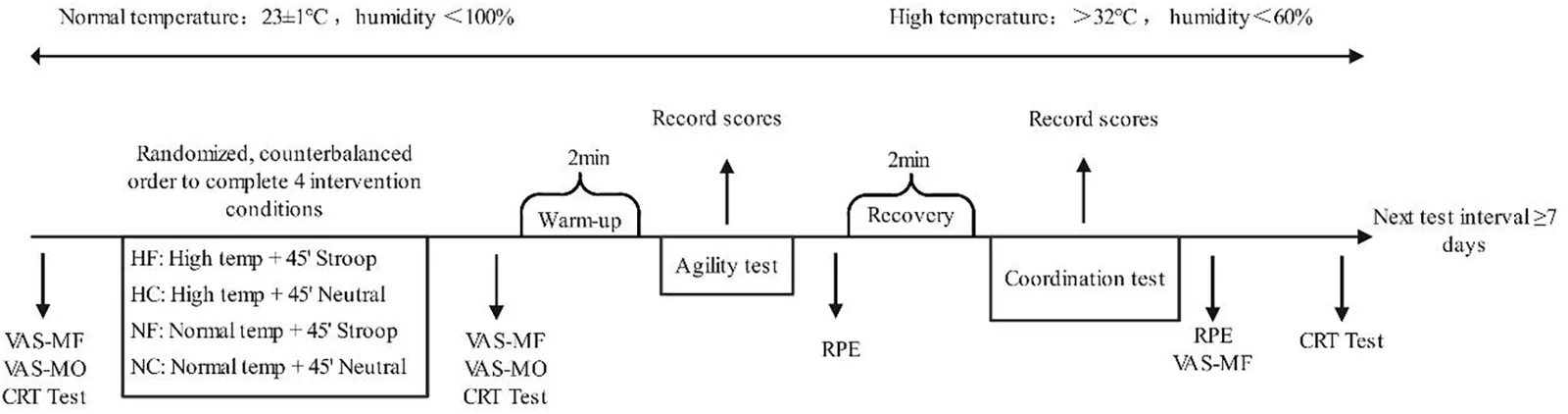
Experimental flowchart. VAS-MF, mental fatigue score; VAS-MO, motivation level; RPE, ratings of perceived exertion; CRT, choice reaction time test.

### 2.3 Intervention

#### 2.3.1 Environmental conditions and timing

To approximate real-world summer training and competition environments as closely as possible,this study was conducted entirely in an enclosed, access-controlled outdoor training ground, using natural summer climatic conditions (Yang et al., 2026). Referring to the methodology of established examples from previous research (Pryor et al., 2021; Wallenberg et al., 2023), appropriate high- and normal-temperature conditions were determined based on temperature announcements from the local meteorological bureau. According to local meteorological records during the experimental period (June–July), the high-temperature condition (afternoon sessions) had an ambient temperature range of 33.14–35.69 °C and a relative humidity range of 21.55–56.80%; the normal-temperature condition (morning sessions) had an ambient temperature range of 24.16–24.99 °C and a relative humidity range of 53.02–98.97%. Due to the natural diurnal variation of humidity in outdoor environments, morning sessions generally had higher relative humidity than afternoon sessions. No artificial humidity regulation was applied in this study to preserve the authenticity of the natural training environment. Specific environmental parameters for each of the four experimental sessions are detailed in **Table 1**. Wet-bulb globe temperature (WBGT), solar radiation, and air velocity were not measured on-site in the present study due to field equipment limitations.The cognitive intervention tasks (Stroop task and neutral control video) were completed in a dedicated, enclosed quiet zone within the training ground to ensure task standardization and minimize external distraction. The warm-up, Illinois Agility Test, and Harre Circuit Test were all conducted on the same artificial turf soccer surface within the training ground, under the corresponding ambient conditions. All participants wore standard soccer cleats for testing, and were required to avoid strenuous physical activity in the 24 hours prior to each session to standardize pre-test physical state.The intervention experiments were conducted during June and July. The normal-temperature and high-temperature intervention sessions were scheduled at different times on the same day, with the normal-temperature sessions taking place from 7:30 to 9:00 AM and the high-temperature sessions from 1:00 to 2:30 PM. All experiments were completed within a 6-week period.

**Table 1:** Environmental parameters of each experimental session.

Session	Condition	Ambient temperature (°C)	Relative humidity (%)
Test 1	Normal temperature (morning, Jun 8)	24.34	64.93
	High temperature (afternoon, Jun 8)	33.96	27.90
Test 2	Normal temperature (morning, Jun 15)	24.99	53.02
	High temperature (afternoon, Jun 15)	35.69	21.55
Test 3	Normal temperature (morning, Jul 14)	24.41	89.80
	High temperature (afternoon, Jul 14)	33.74	51.52
Test4	Normal temperature (morning, Jul 28)	24.16	98.97
	High temperature (afternoon, Jul 28)	33.14	56.80

#### 2.3.2 MF induction protocol

The induction of MF in this study was based on a Stroop task administered via a mobile device (tablet). Sustained elicitation and recruitment of response inhibition and cognitive control (≥30 min) can effectively induce an MF state, as this task is a classic paradigm for evoking cognitive conflict and continuously engaging executive functions—particularly response inhibition and cognitive control. Multiple studies have confirmed the efficacy of this task in inducing MF (Badin et al., 2016; Kunrath et al., 2020; Trecroci et al., 2020). Referring to the established protocol developed in previous studies (Smith et al., 2015; Smith et al., 2017), and taking into account the practical requirement of conducting this study under natural high-temperature conditions, a Stroop task application (App) was employed for the induction to ensure both feasibility and standardization of the intervention protocol (Bajaj et al., 2013), with the task duration set at 45 minutes. The specific procedures were as follows: participants independently operated the tablet device installed with the Stroop task App in the dedicated, enclosed quiet zone of the training ground. During the task, four color words—”red,” “green,” “blue,” and “yellow”—were presented on the screen in random order, with the font color of each word being either congruent or incongruent with its semantic meaning (with incongruent trials accounting for 50% of all trials). Participants were instructed to ignore the semantic meaning of the words and to respond as quickly and accurately as possible based solely on the actual color of the text. Throughout the task, six trained researchers were present to supervise the participants, ensuring that they maintained a high level of cognitive engagement during the entire intervention period.

#### 2.3.3 Control group protocol

For the control condition, participants watched a 45-minute neutral video documentary, *Tale of Lake*, which matched the duration of the MF induction task. This documentary features tranquil content and a slow-paced narration. Previous research has confirmed that it effectively maintains participants’ emotional stability without inducing significant cognitive load or MF, making it a suitable control stimulus (Badin et al., 2016). To ensure consistency across experimental conditions, participants in the control groups (HC and NC) used the same tablet devices as those in the MF induction groups (HF and NF) and watched the documentary independently in the same dedicated, enclosed quiet zone of the training ground. Throughout the session, six trained researchers were also present to supervise the participants, ensuring that they remained awake and attentive to the video.

### 2.4 Agility test

Change-of-direction speed, a core component of agility, was assessed using the Illinois Agility Test (IGT), a widely adopted pre-planned agility measure in soccer populations (Amiri-Khorasani et al., 2010; Aouadi et al., 2011; Kutlu et al., 2017). This test employs a fixed, pre-determined running route without unpredictable external stimuli, and thus primarily reflects pre-planned change-of-direction performance rather than reactive agility.The layout of the testing area and the running route are illustrated in **Figure 3**. The specific procedures were as follows: participants assumed a standing start position, bending forward behind the starting line. Upon the verbal command “Go,” participants sprinted at maximal speed, weaving around the cones along the designated route, and finally crossing the finish line. Participants were required to circle around the outside of each cone during the run and were not allowed to knock over any cones. Each participant completed two official trials, with a 2-minute rest interval between trials, and the average of the two trials was used as the final data for analysis. All timing was administered by two rigorously trained and experienced researchers, who served as fixed assessors across all experimental sessions. For each single trial, the completion times recorded by the two assessors were averaged to serve as the final score of that trial. This dual-assessor averaging approach is a standardized procedure widely used in sport performance tests to minimize systematic error of manual timing and ensure measurement reliability. Previous studies have demonstrated that, when used as a measurement tool for tests similar to those in the present study, a stopwatch offers high precision and strong correlation with photoelectric timing gates (>0.95) (Chen et al., 2021).Therefore, a stopwatch was used to record the completion time of the entire course, with accuracy to 0.01 seconds. To eliminate the potential influence of changes in reaction time (as MF may prolong participants’ response to the “Go” command) caused by the experimental variables (ambient temperature and MF induction) on agility performance, the time from the moment the participant left the starting line to the moment they crossed the finish line was recorded as the test score.

**Figure 3:**
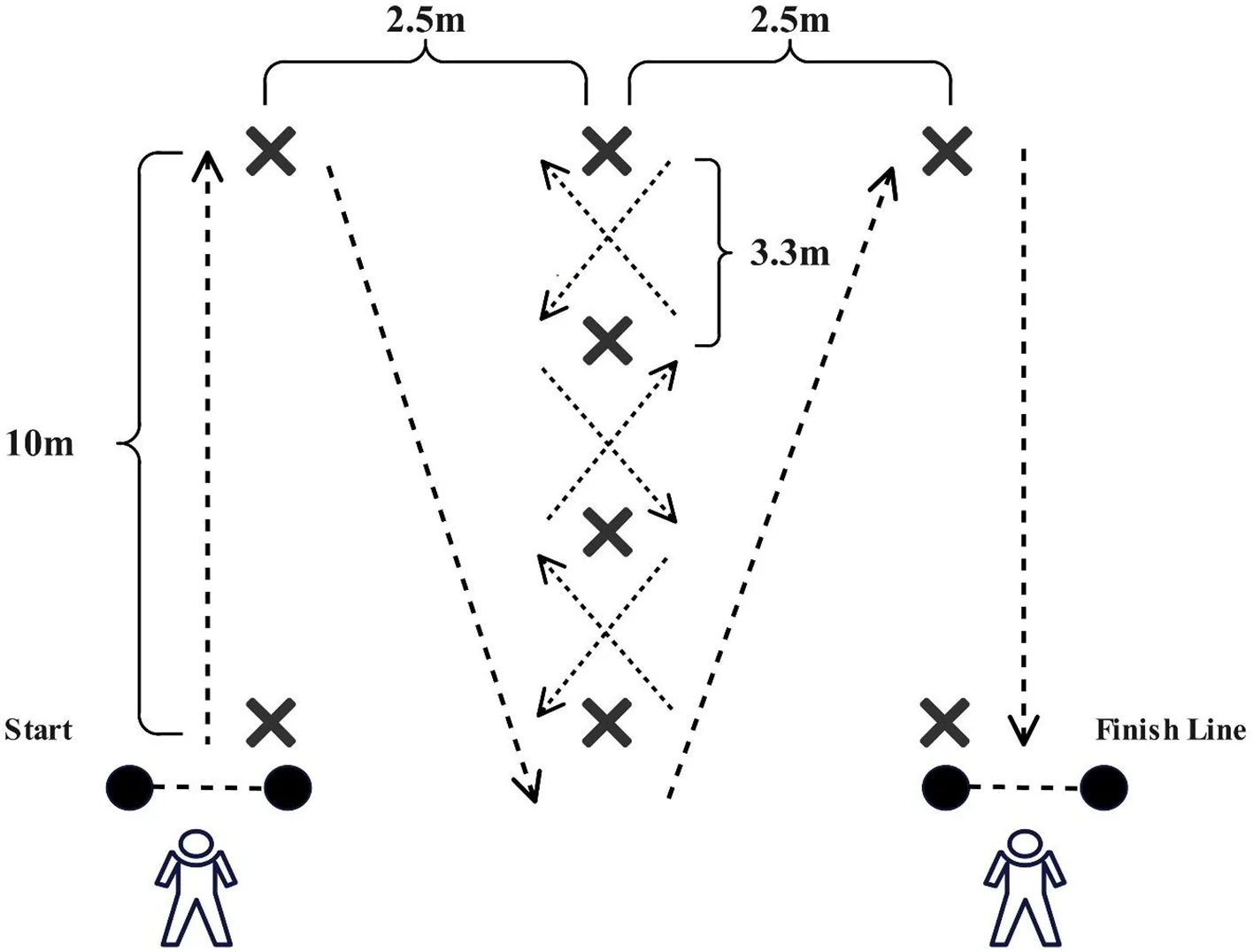
Layout and running route of the Illinois agility test.

### 2.5 Coordination test

Coordination was assessed using the revised version of the Harre Circuit Test (HCT) as employed in the studies by Trecroci et al. (2015). The layout of the testing area and the running route are illustrated in **Figure 4**. The specific procedures were as follows: participants stood behind the starting line. Upon the verbal command “Go,” participants sprinted forward from the start, crossed over a mat, and approached the central cone at maximal speed. They then executed a rapid change of direction around the outside of the central cone, circled around the outside of pole No. 1, quickly returned to the central cone, changed direction again to circle around poles No. 2 and No. 3, and finally ran back to the finish line. Each participant completed two official trials, with a 2-minute rest interval between trials, and the average of the two trials was used as the final data for analysis. The time to complete the entire course was recorded by the same two fixed trained assessors using a stopwatch, with the average of the two recordings taken as the final score for each trial, to the nearest 0.01 seconds.

**Figure 4:**
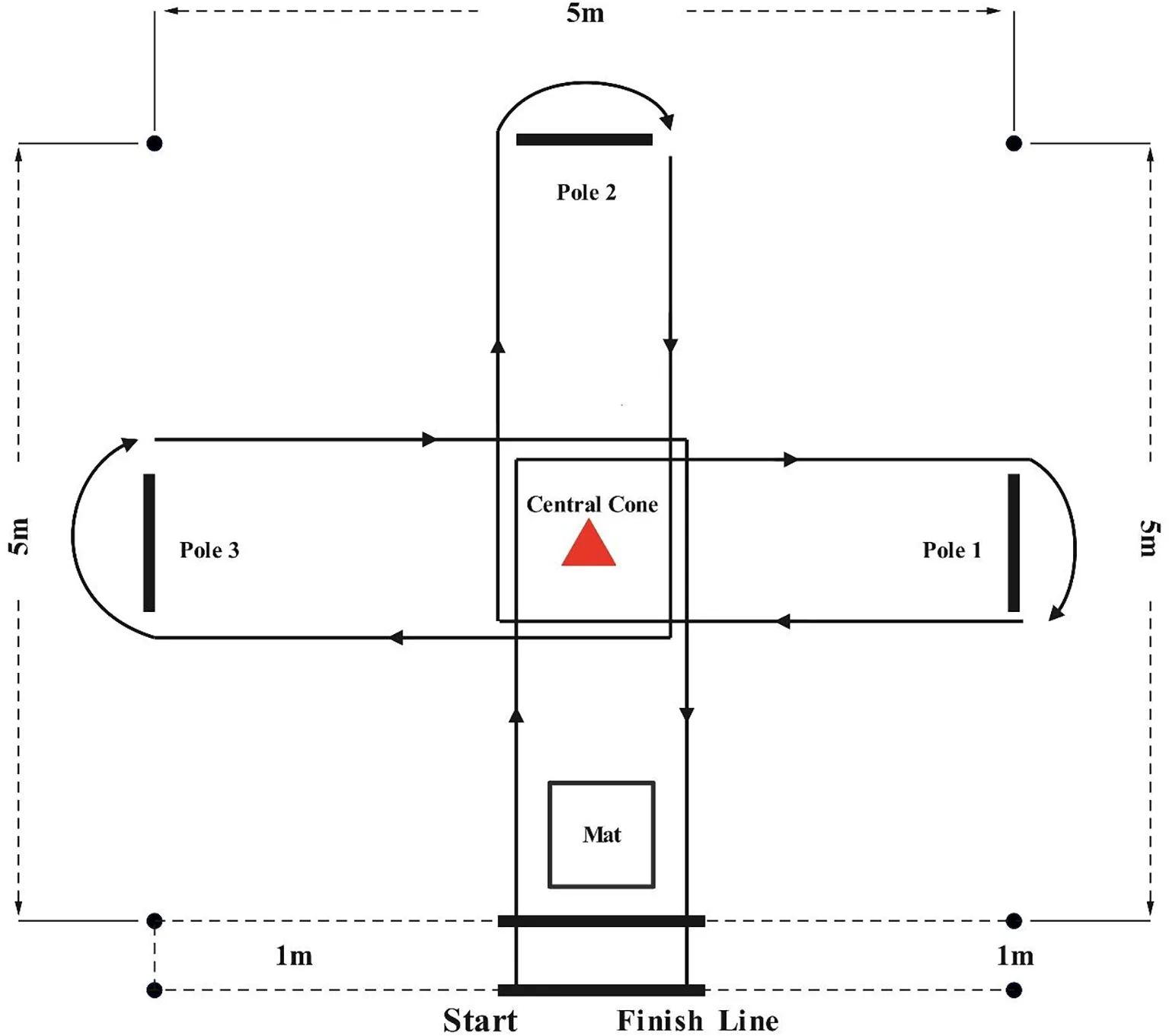
Layout and running route of the Harre circuit test.

### 2.6 Tools

To evaluate the effects of the induction protocols on participants’ psychological state, this study employed subjective scales that are commonly used in the literature to quantify the degree of MF, motivation level, and perceived exertion (Ding et al., 2024; Daneshgar-Pironneau et al., 2025; Hemmat et al., 2025). All scales were administered by uniformly trained researchers at specific time points during each experimental session (pre-intervention, immediately post-intervention, and immediately after the physical tests) to ensure standardization and consistency of data collection. In addition, choice reaction time was adopted as an objective cognitive indicator. According to existing literature (Van Cutsem et al., 2017b; Pageaux and Lepers, 2018; Gattoni et al., 2021), the primary criterion for successful MF induction is that the experimental group exhibits significantly higher VAS-MF scores than the control group after the intervention, along with a significantly greater pre-to-post increase in VAS-MF scores in the experimental group compared to the control group; prolonged reaction time serves as a secondary criterion for successful MF induction.

#### 2.6.1 Subjective measurement instruments

Visual Analog Scale for Mental Fatigue (VAS-MF) and Visual Analog Scale for Motivation (VAS-MO): A 100-mm Visual Analog Scale was used to assess participants’ degree of mental fatigue and motivation level, respectively. The scale consists of a 100-mm straight line with the anchors “Not at all” and “Extreme/Maximum” at each end. For assessing MF, participants were instructed to mark on the line according to the extent to which they currently felt “mentally exhausted, fatigued, and sluggish in thinking.” For assessing motivation, they were instructed to mark according to the extent of their “willingness and drive to participate in the subsequent physical tests.” The researchers measured the distance (in mm) from the origin to the mark as the score.

Ratings of Perceived Exertion Scale (RPE): The Borg 6–20 point RPE scale was used to assess participants’ perceived exertion following the physical tests (Borg, 2001). This scale categorizes subjective feelings during exercise into 15 levels, ranging from “6 (Very, very light)” to “20 (Exhausted).” Immediately after each physical test (the Illinois Agility Test and the Harre Circuit Test), participants were asked to indicate or report the corresponding level based on their overall bodily sensations during the test.

#### 2.6.2 Objective measurement instruments

A multiple reaction time apparatus (Model BD-II-509B) was used to collect participants’ reaction time data. The Choice Reaction Time (CRT) test was selected as the objective indicator for assessing MF status. This test required participants to respond as quickly and accurately as possible to specific visual stimuli randomly presented on the screen (e.g., signals of different directions or colors) according to predetermined rules. The testing program automatically recorded the average reaction time (in ms) while excluding erroneous responses and outliers, with 20 trials per session. In this study, the CRT test was administered at three time points: pre-intervention, immediately post-intervention, and after all physical tests were completed. This objective measure effectively circumvented the bias inherent in subjective reports and provided psychophysiological evidence for the presence of MF.

### 2.7 Statistical analysis

Statistical analyses were performed using SPSS 26.0 software. All continuous data were confirmed to follow a normal distribution by the Shapiro-Wilk test and were presented as mean ± standard deviation (M ± SD). To examine the effects of high ambient temperature and MF on the outcome variables, repeated-measures ANOVA was applied to the primary outcome measures. Specifically, a three-way repeated-measures ANOVA was used for VAS-MF scores, VAS-MO scores, CRT, and RPE, with ambient temperature (high vs. normal), MF induction (yes vs. no), and time (e.g., pre-intervention, post-intervention, after physical tests) as within-subjects factors. For agility and coordination test performance, a two-way repeated-measures ANOVA was employed, with ambient temperature (high vs. normal) and MF induction (yes vs. no) as the two within-subjects factors.

When the sphericity assumption was violated, the Greenhouse-Geisser correction was applied. The repeated-measures ANOVA primarily focused on the main effects of each factor and their interactions. When a significant interaction was detected, simple effects analysis was further conducted, with Bonferroni correction applied for multiple comparisons to delineate the specific patterns. To verify the effectiveness of the MF induction protocol, the main effect of MF on VAS-MF scores was used as the primary criterion for determining whether the induction was successful. All statistical tests were two-tailed, with the significance level set at α = 0.05. For significant results, partial eta-squared (η²p) was reported as the effect size for main effects and interactions, and Cohen’s dz (within-subjects Cohen’s d) was used for all pairwise comparisons and simple effects analyses to match the repeated-measures crossover design of the study (Lakens, 2013). The criteria for interpreting effect sizes were according to established effect size interpretation criteria of Smith et al. (2016): η²p values of ≥0.01, ≥0.06, and ≥0.14 were considered small, medium, and large, respectively; dz values of ≥0.2, ≥0.5, and ≥0.8 represented small, medium, and large effects, respectively.

### 2.8 Reporting guideline statement

This randomized crossover study was conducted and reported in full accordance with the Consolidated Standards of Reporting Trials (CONSORT) 2025 guidelines and its official extension for crossover trials. A completed CONSORT checklist is provided as an additional Supplementary File with this revised manuscript.

## 3 Results

### 3.1 MF induction status and analysis of related indicators

The 45-minute Stroop task successfully induced an MF state under both temperature conditions (**Table 2**). The repeated-measures ANOVA revealed significant main effects of both ambient temperature and MF induction on VAS-MF scores, while the three-way Temperature × MF × Time interaction did not reach statistical significance (p = 0.413) (**Table 4**). This indicates that high ambient temperature elevated the overall level of subjective fatigue perception, but did not significantly amplify the magnitude of fatigue induced by the Stroop task.The repeated-measures ANOVA revealed a significant MF × Time interaction for VAS-MF scores(F_(1.750,31.498)_=55.599, *p* < 0.001,η²_p_=0.755). Simple effects analysis indicated that, under both temperature conditions, the MF induction groups exhibited a significant increase in VAS-MF scores from baseline to post-intervention, although the magnitude of increase differed: the effect size for the increase under high-temperature conditions (d_z_=-6.564, 95% CI = -8.852 ~ -4.276)was larger than that under normal-temperature conditions(d_z_=-2.599, 95% CI=-3.607 ~ -1.591 ). More critically, the Temperature × Time interaction was also significant(F_(1.684,30.314)_=15.607,*p* < 0.001,η²_p_=0.464). Immediately post-intervention, the high-temperature MF condition (HF) reached the peak VAS-MF score (80.47 ± 4.78), which was significantly higher than that of the normal-temperature MF group (NF, 68.22 ± 10.50;P<0.001, d_z_= 2.818, 95% CI = 1.743~3.892);Under both high- and normal-temperature conditions, the 45-minute Stroop task successfully induced significant MF (HF vs NC: d_z_=0.910, 95% CI = 0.336~1.483;NFvs NC:d_z_= 1.129, 95% CI = 0.512~1.746;both P<0.001), indicating that both the cognitive task and high ambient temperature contributed independently to higher subjective fatigue levels.VAS-MF scores in all conditions showed some relief after the physical tests; however, the levels in the MF induction groups remained significantly higher than those in their respective control groups (high-temperature: d_z_ = 0.788, 95% CI = 0.236~1.339; normal-temperature: d_z_ = 0.803, 95% CI = 0.249~1.357;both P<0.001), and remained far above baseline levels, exhibiting a persistent characteristic. This indicates that the MF state continues to pose a sustained threat to subsequent physical performance.

**Table 2:** VAS-MF and choice reaction time (CRT) at each time point under different experimental conditions.

Measure	Ambient temperature	MF induction	Group	Pre-intervention	Post-intervention	Post-physical tests
VAS-MF(mm)	High	Yes	HF	23.05 ± 7.70	80.47 ± 4.78	73.74 ± 8.97
	High	No	HC	22.29 ± 6.74	46.32 ± 12.58	43.89 ± 18.74
	Normal	Yes	NF	23.78 ± 13.11	68.22 ± 10.50	60.9 ± 17.13
	Normal	No	NC	25.83 ± 10.15	29.34 ± 9.91	27.68 ± 8.85
CRT(s)	High	Yes	HF	0.5711 ± 0.1131	0.7434 ± 0.098	0.6276 ± 0.1343
	High	No	HC	0.5820 ± 0.1043	0.6008 ± 0.1224	0.5698 ± 0.1264
	Normal	Yes	NF	0.5939 ± 0.1526	0.6630 ± 0.2499	0.5982 ± 0.1421
	Normal	No	NC	0.5893 ± 0.1251	0.5865 ± 0.1659	0.5447 ± 0.1273

The change pattern of CRT further corroborated the acute impairing effect of MF on cognitive function, and suggested that such cognitive decline is reversible, recovering to baseline levels after physical exertion, which stood in contrast to the persistent nature of subjective MF perception (**Tables 2**, **3**). The MF × Time interaction for CRT was significant(*F*_(1.562,28.120)_=4.897, *p* = 0.021,η²_p_=0.214). Post-intervention, the CRT of the MF induction groups was significantly longer than that of the control groups(*p* = 0.001, d_z_ = 0.935), indicating a decline in response speed of executive function. However, in contrast to the sustained elevation of subjective perception, the CRT of the MF induction groups returned to baseline levels after the physical tests, with no significant difference from the control groups (p = 0.067). This dissociation in the recovery trajectories between subjective and objective measures suggests that the transient decline in CRT may primarily reflect the depletion of cognitive resources, whereas the sustained elevation of VAS-MF captures a more complex integrative state of physical and mental fatigue.

**Table 3:** Repeated-measures ANOVA results for choice reaction time (CRT).

Source	F	P	η²p
Ambient temperature (T)	1.429	0.247	0.074
MF	9.544	***0.006***	0.347
Time (Ti)	5.164	***0.014***	0.223
T×MF	0.260	0.617	0.014
T×Ti	1.185	0.315	0.062
MF×Ti	4.897	***0.021***	0.214
T×MF×Ti	0.696	0.497	0.037

Bold values indicate statistically significant effects at the p < 0.05 level in the repeated‑measures ANOVA.

**Table 4:** Repeated-measures ANOVA results for VAS-MF scores.

Source	F	P	η²p
Ambient temperature (T)	44.667	***<0.01***	0.713
MF	233.934	***<0.01***	0.929
Time (Ti)	162.841	***<0.01***	0.900
T×MF	0.276	0.606	0.015
T×Ti	15.607	***<0.01***	0.464
MF×Ti	55.599	***<0.01***	0.755
T×MF×Ti	0.864	0.413	0.046

Bold values indicate statistically significant effects at the p < 0.05 level in the repeated‑measures ANOVA.

### 3.2 Agility and coordination test results

The physical test results revealed that the effects of high ambient temperature and MF on agility and coordination differed (**Figures 5**, **6**).

**Figure 5:**
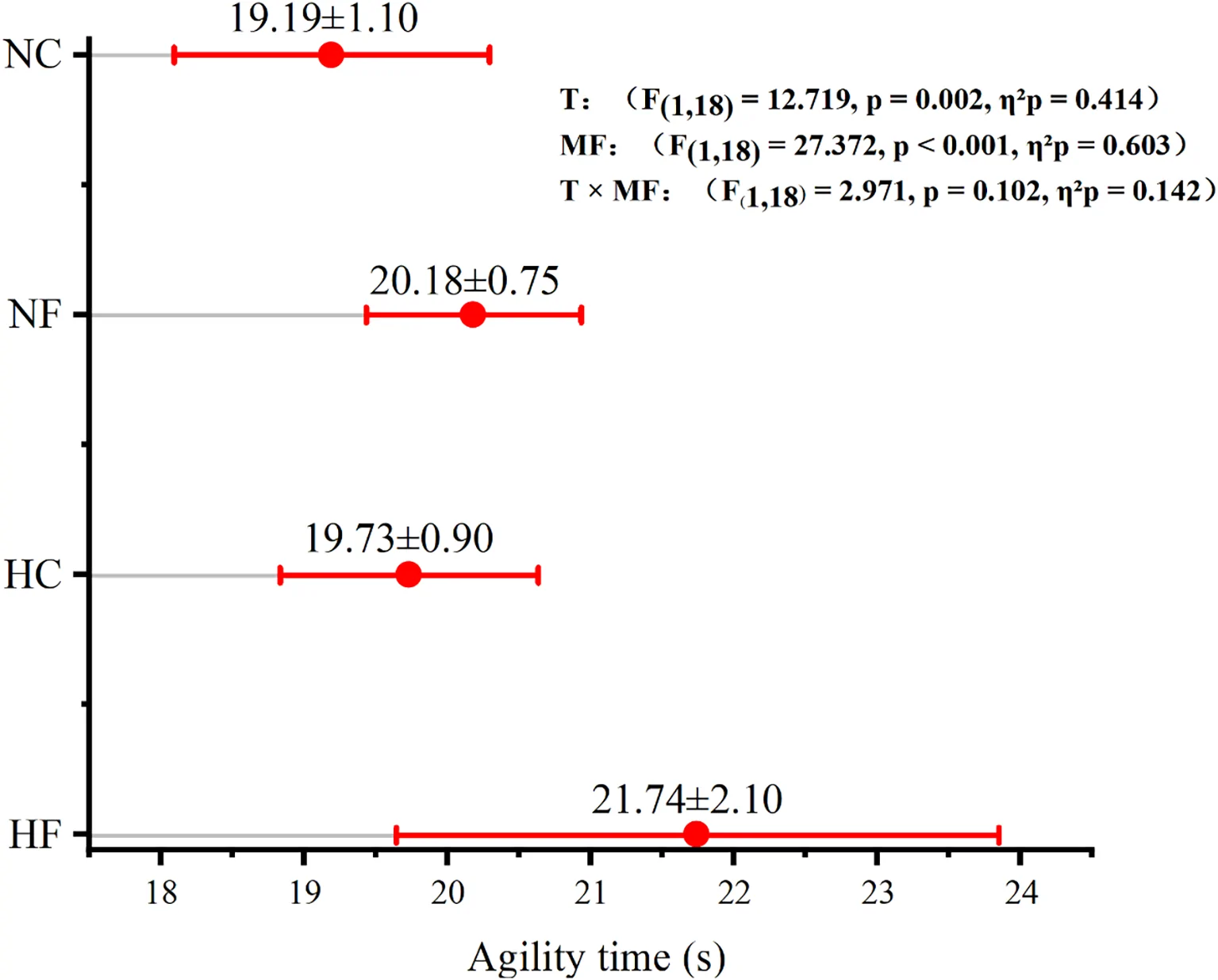
Agility test performance across conditions.

**Figure 6:**
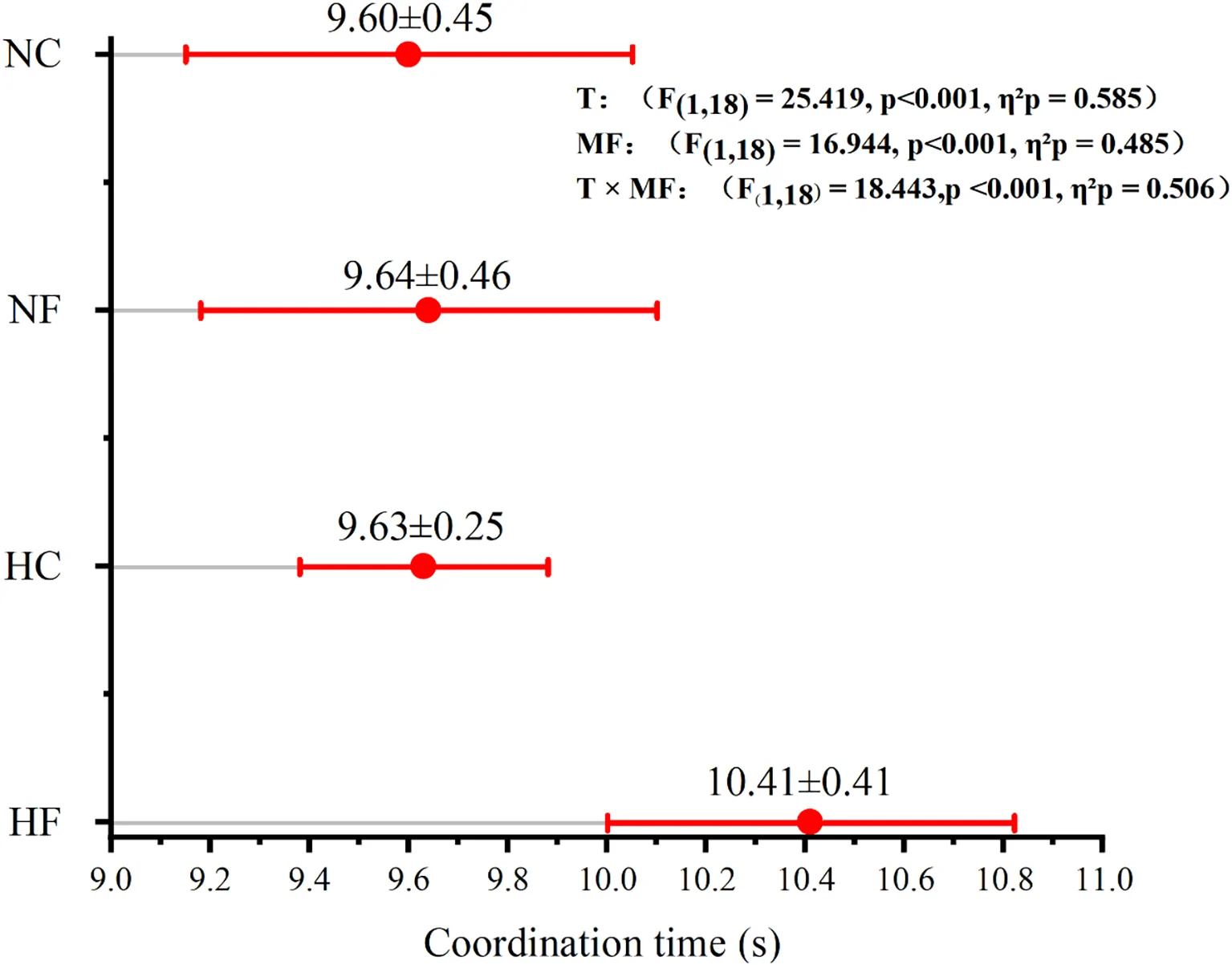
Coordination test performance across conditions.

For agility, the effects of the two factors were independent and additive. The ANOVA showed that the main effects of both MF(F_(1,18)_=27.372,*p* < 0.001,η²_p_=0.603) and high ambient temperature(F_(1,18)_=12.719,*p* = 0.002,η²_p_=0.414)were significant, whereas the Temperature × MF(F_(1,18)_=2.971,*P* = 0.102,η²_p_=0.142) interaction did not reach statistical significance. The partial eta-squared for the interaction term was 0.142, which falls into the range of a large effect size according to our predefined criteria, suggesting that the lack of significance may be partly related to insufficient statistical power with the current sample size. The pattern of results was most consistent with an independent additive effect of the two factors on agility.Simple effects analysis revealed that,under high-temperature conditions, the MF group performed significantly slower in the agility test than the control group(*d_z_ = 0.676, 95% CI = 0.142~1.210*, *p* = 0.003);under normal-temperature conditions, the MF condition showed a trend toward slower agility test performance compared with the control condition, but this difference did not reach statistical significance(*d_z_* = 0.461, 95% CI = −0.046~0.968, P = 0.011);under non-MF conditions, the high-temperature group performed significantly worse than the normal-temperature group(*d_z_* = 0.654, 95% CI = 0.123~1.185, P = 0.015);and under MF conditions, the high-temperature group showed a further significant decline in performance(*d_z_* = 0.981, 95% CI = 0.395~1.568, P = 0.002). These findings indicate that, regardless of ambient temperature, MF impairs agility; conversely, regardless of MF status, high temperature significantly prolongs test completion time.

**Figure 7:**
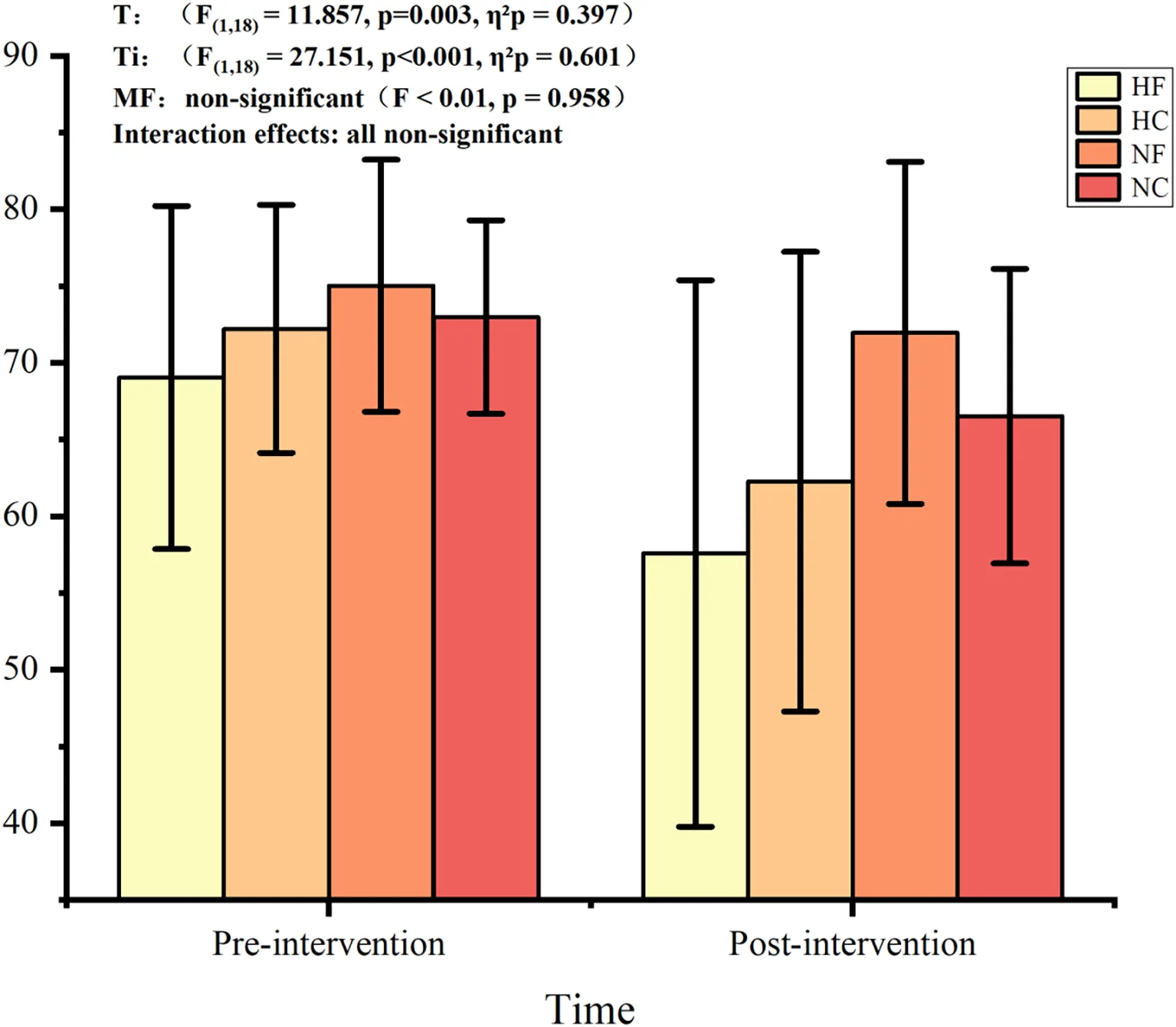
Comparison of VAS-MO scores pre- and post-intervention across groups.

For coordination, the main finding of this study was a significant Temperature × MF interaction effect on performance.The ANOVA revealed a significant Temperature × MF interaction(F_(1,18)_=18.443,*p* < 0.001,η²_p_=0.506). Simple effects analysis further clarified the nature of this interaction: under normal-temperature conditions, there was no significant difference in coordination performance between the MF induction group and the control group(*p* = 0.751, d_z_ = 0.072, 95% CI = −0.411~0.555), with a negligible effect size, indicating that MF alone did not pose a threat; similarly, under non-MF conditions, performance in the high-temperature condition did not significantly decline(*p* = 0.757, d_z_ = 0.075, 95% CI = −0.408~0.557), with the effect size remaining at a low level. However, when both factors coexisted—that is, the MF induction group under high-temperature conditions—their coordination performance was significantly worse than that of the high-temperature control group (*p* < 0.001, d_z_ = 1.247, 95% CI = 0.604~1.889) and the normal-temperature MF group(*p* < 0.001, d_z_ = 1.498, 95% CI = 0.796~2.200). This indicates that the impairing effect of MF on coordination is dependent on high ambient temperature; the negative effect that was masked under normal-temperature conditions was fully “triggered” under the physiological stress of heat.

**Figure 8:**
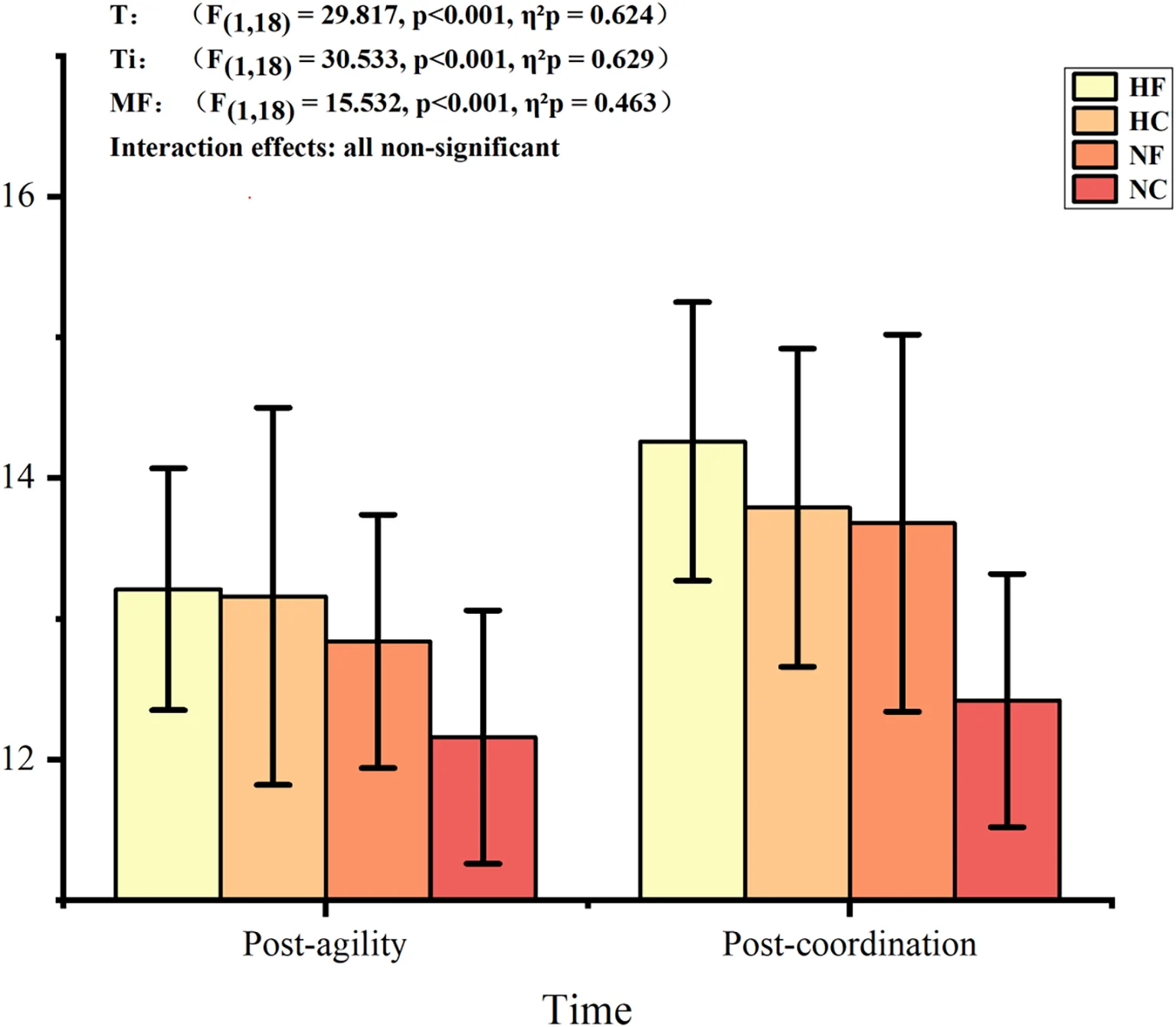
Comparison of RPE after agility and coordination tests across groups.

### 3.3 Other indicator analyses

Changes in motivation level (VAS-MO) were primarily influenced by ambient temperature. The ANOVA revealed that the main effect of ambient temperature was significant(*F*_(1,18)_=11.857,*p* = 0.003,η²_p_=0.397), whereas the main effect of MF induction was not(*F* < 0.01,*p* = 0.958), and all interaction effects were non-significant (*p* > 0.05). Simple effects analysis showed that the magnitude of reduction in motivation induced by high temperature was greater under MF conditions(*d_z_ =-0.183, 95% CI=-0.669~0.303*), than under control conditions(*d_z_ =0.541, 95% CI = 0.025~1.058*). This suggests that, compared with cognitive load, the physiological stress of high ambient temperature is the predominant factor undermining athletes’ willingness to engage in subsequent physical activity.

Ratings of Perceived Exertion (RPE) were independently influenced by both high temperature and MF. The ANOVA showed that the main effect of ambient temperature was significant (*F*_(1,18)_=29.817,p<0.001,η²_p_=0.624);the main effect of MF was also significant(*F*_(1,18)_=15.532,p=0.001,η²_p_=0.463); and all interaction effects were non-significant (all p > 0.05). This indicates that, under the present conditions, the perception of “effort” experienced by athletes during competition or training can be independently induced by high ambient temperature or by MF state. When both factors coexist, athletes experience a doubly amplified sense of effort arising from both external environmental and internal psychological stresses (**Figures 7**, **8**).

## 4 Discussion

### 4.1 Induction effects of MF under high ambient temperature and its modulating role

The primary premise of this study was to verify the cross-condition validity of the MF induction protocol. The results demonstrated that the 45-minute Stroop task successfully induced an MF state in female soccer players under both normal- and high-temperature conditions, and further revealed that high ambient temperature exacerbated this process. These mechanisms are not directly tested in the present study. Based on existing literature, three potential explanations have been proposed for the development of MF: First, the development of MF may be associated with enhanced alpha and theta wave activity in the prefrontal cortex (Trecroci et al., 2015; Van Cutsem et al., 2017a; Franco-Alvarenga et al., 2019; Brietzke et al., 2020). Second, Ishii (Ishii et al., 2014) systematically explored the possible mechanisms of MF from a macro-level perspective, attributing its occurrence to insufficient activation of the mental facilitation system, enhanced activity of the mental suppression system, or a combination of both (i.e., concurrent weakening of the former and strengthening of the latter). Third, the emergence of MF may be closely related to the reduction of locally available energy substrates in the anterior cingulate cortex, leading to the accumulation of extracellular adenosine concentration (Van Cutsem et al., 2017b; Martin et al., 2018; Smith et al., 2018).

The success of MF induction was reflected in the changes in subjective (VAS-MF, as the primary criterion) and objective (CRT) indicators before and after the intervention (Van Cutsem et al., 2017b; Gattoni et al., 2021). For subjective measures, there was no significant difference in baseline VAS-MF scores between the MF induction groups and control groups (*p* = 0.776). However, after the intervention, VAS-MF scores in the induction groups increased significantly from baseline (*p* < 0.001), with a substantially larger magnitude of increase (d = -4.814) compared to the natural fluctuation observed in the control groups (d = -1.602). Moreover, post-intervention VAS-MF scores in the induction groups were significantly higher than those in the control groups (*p* < 0.001, d = 5.362), consistent with the consensual criteria for successful MF induction in the field (Smith et al., 2016; Van Cutsem et al., 2017b; Martin et al., 2018). For objective measures, the induction groups also exhibited a significant prolongation of CRT after the intervention (*p* = 0.003), which differed significantly from the control groups (*p* = 0.001). This provided physiological evidence of cognitive performance decline, corroborating the induction effect of MF and supporting, to some extent, the findings of Migliaccio et al. (2022).

Meanwhile, the present study found that high ambient temperature did not serve as a neutral background condition, but was independently associated with higher overall levels of subjective fatigue perception.The ANOVA revealed that the main effect of ambient temperature on VAS-MF was highly significant (*F*_(1,18)_=44.667,*P* < 0.001,η²p=0.713),with a large effect size. At the post-intervention time point, the VAS-MF score in the high-temperature groups was approximately 12 mm higher than that in the normal-temperature groups (d = 2.149), indicating that athletes in the high-temperature environment experienced a stronger subjective feeling of MF after completing the same cognitive task. Given that the three-way Temperature × MF × Time interaction was not significant, our data support that high temperature elevates the baseline and overall level of subjective fatigue, rather than specifically amplifying the fatigue-inducing effect of the Stroop task itself.This modulating effect may stem from the multiple physiological and psychological stresses induced by high temperature—such as thermal sensation and increased cardiovascular load—which overlap with the cognitive load from the task, jointly accelerating the accumulation of central subjective fatigue (Bligh and Johnson, 1973; Maughan, 2010).

Notably, the VAS-MF scores measured after the physical tests indicated that once the MF state was established, it exhibited a persistent nature. Despite the warm-up and the two physical tests, the VAS-MF scores of the MF induction groups remained significantly higher than those of the control groups, with a large effect size (d = 2.919). Comparison with their own baseline levels before the intervention further revealed that, although the fatigue levels in the induction groups had alleviated from the post-intervention peak, they were still far from returning to the initial state, with VAS-MF scores significantly higher than pre-intervention baseline (p < 0.001, d = -3.306). This indicates that MF is difficult to dissipate naturally within a short period, as evidenced by comparisons of VAS-MF scores immediately after the sport-specific coordination test with pre-task baseline levels and with those of the control group—a finding consistent with multiple previous studies (Brownsberger et al., 2013; Smith et al., 2025). In contrast, the objective CRT measure recovered relatively quickly after the physical tests. This dissociation suggests that VAS-MF may not merely capture the depletion of cognitive resources, but rather integrate emotional, interoceptive, and physical discomfort signals. Consequently, under the complex stress of concurrent high temperature and fatigue, VAS-MF exhibited a more enduring warning signature. This finding implies that brief rest or warm-up activities during actual competition or training may be insufficient to eliminate athletes’ subjective feelings of fatigue.

### 4.2 Effects of high ambient temperature and MF on agility

Morral (Morral-Yepes et al., 2022) defined agility as rapid whole-body movement in response to changes in speed or direction triggered by a stimulus. A broad construct encompassing both reactive agility (responding to unpredictable external events) and pre-planned change-of-direction performance. The IGT used in this study primarily assesses the latter, which still relies on cognitive resources including sustained attention, motor control and movement timing regulation.Agility is also one of the essential abilities that soccer players must possess during matches (Pojskic et al., 2018). Existing research has confirmed that MF impairs athletes’ cognitive functioning, with affected individuals showing decreased accuracy, prolonged reaction times, and compromised decision-making ability on cognitive tasks (Smith et al., 2016; Pires et al., 2018; Fortes et al., 2023). Meanwhile, studies have demonstrated that high ambient temperature significantly affects human cognitive function. Passive heat exposure in a hot environment likewise leads to prolonged visual attention reaction time and declined cognitive performance (Jiang et al., 2013). Research has shown that when core body temperature rises to 37.8 °C in a hot environment, cerebral blood flow and cerebral oxygenation decrease, resulting in reduced oxygen supply, along with neurotransmitter dysregulation and a reduction in total cognitive resources (Douzi et al., 2020). Thus, both MF and high ambient temperature can indirectly influence agility performance via cognitive pathways.

However, research on whether MF affects athletes’ agility performance is relatively scarce and somewhat inconsistent. Weerakkody et al. (2021) investigated the effects of MF on change-of-direction ability in 25 male amateur rugby players and found no significant difference between the MF group (30-min Stroop task) and the control group (30-min neutral documentary), indicating that MF did not affect the players’ change-of-direction performance. The findings of the present study differ from the above conclusions, as both the main effects of ambient temperature and MF were significant, while their interaction did not reach statistical significance, indicating that MF and high ambient temperature are two independent factors impairing agility performance, thereby supporting Hypothesis 1. This discrepancy in research findings, as well as the reasons why MF affected agility in the present study, can be attributed primarily to the following two aspects:1)MF exerted a significant negative effect on cognitive function, and cognitive factors are among the determinants of athletes’ agility performance. By impairing cognitive abilities, MF indirectly affected performance in the agility test. After the Stroop task induced MF, athletes showed elevated MF scores and prolonged reaction times, and those in an MF state typically also exhibited concurrent significant declines in decision-making ability (Smith et al., 2016). Soccer demands a high level of cognitive function and capacity from players, requiring sustained high concentration during technical training and matches, rapid information capture and processing in a fast-paced and ever-changing pitch environment, and prompt decision-making (Smith et al., 2018). Thus, MF triggered a cascade of negative effects on cognitive function, which in turn led to impairments in agility—a result reflected in the significant main effect of MF.2)the magnitude of the effect of MF on agility performance is related to the athletes’ competitive level. Although the male rugby players recruited by Weerakkody et al. (2021) were unaffected by MF in change-of-direction and agility tests, this may be because their agility movements had already formed highly automated neural pathways with low reliance on higher-order cognitive centers, thereby limiting the impact of MF-induced cognitive decline on agility performance. In contrast, the female collegiate soccer players recruited in the present study relied more heavily on cognitive resources to complete the agility test, with a relatively lower degree of movement automation, making them more susceptible to the effects of MF and consequently more vulnerable to its negative impact. The cognitive decline induced by MF had a limited effect on the former group’s agility test performance, but exerted an overall effect on the performance of the present study’s participants in the agility test. Therefore, MF produced a significant negative effect on the agility performance of female soccer players.

Meanwhile, the results showed that agility performance was significantly poorer under the high-temperature condition than under the normal-temperature condition. Although ambient temperature is the most plausible driver of this difference, we acknowledge that time-of-day variation may also have played a contributing role. This observed impairment may be explained by two interrelated potential pathways:1)high ambient temperature exerts a significant negative effect on cognitive function, which in turn indirectly affects agility test performance. A meta-analysis (Pilcher et al., 2002), demonstrated that the relationship between ambient temperature and cognitive performance follows an inverted U-shaped curve, with cognitive performance declining significantly under high-temperature conditions (above 32.2 °C) and peaking under normal-temperature conditions (21.6 °C), where cognitive performance is optimal. This explains why the normal-temperature group exhibited peak cognitive performance prior to testing, whereas the high-temperature group had already experienced an overall decline in cognitive capacity. Another meta-analysis further elucidated that cognitive performance decreases by 7.97% in environments above 26.8 °C, and that the more attention-demanding the complex and perceptual tasks, the more pronounced the detrimental effects of high temperature (Yeganeh et al., 2018). Furthermore, response inhibition—a fundamental component of cognitive function referring to the ability to suppress unnecessary or inappropriate responses in a changing environment (Coehoorn et al., 2024) —has been shown to be impaired by heat exposure. Ward et al. (2021) found that 3 hours of heat exposure (34 °C) led to impaired response inhibition and consequent cognitive decline. Similarly, Malcolm et al. (2018) investigated the effects of heat exposure on response inhibition and reported that 1 hour of passive heat exposure at 40 °C prolonged subsequent Stroop task reaction times and reduced response inhibition function. Collectively, these findings indicate that high ambient temperature induced significant cognitive performance decrements, which in turn affected agility test performance that requires substantial cognitive resource engagement.2)high ambient temperature produces negative effects on both physiological and psychological aspects of the human body. Exercise in a hot or thermally stressful environment induces heat stress—a systemic integrative response manifested in both physiological and psychological dimensions, which constitutes a primary cause of performance decrements in hot environments (Sawka et al., 2011; Cheshire, 2016; Jung et al., 2021; Fang et al., 2023). Physiologically, high temperature slows nerve conduction velocity, prolongs the muscle contraction–relaxation cycle, and accelerates lactate accumulation, thereby reducing muscle explosive power (Atha, 2013; Cheshire, 2016; Jung et al., 2021). Psychologically, athletes may experience exercise-related anxiety and psychological stress in response to the hot environment, leading to decreased competitive motivation and volitional quality (Cheung, 2007; Fang et al., 2023). Thus, the dual physiological and psychological burden imposed by high ambient temperature constrained the participants’ ability to perform optimally in the agility test.

In summary, the current study did not detect a statistically significant interaction between MF and high ambient temperature on agility performance, and the pattern of findings supports an independent additive effect model. We acknowledge that the interaction term had a moderately large effect size, and we cannot rule out the possibility of a weak interaction that was not detected due to limited statistical power.

### 4.3 Effects of high ambient temperature and MF on coordination

Coordination is the ability of the body’s various organs and systems to cooperate with each other in both temporal and spatial dimensions during movement or the execution of a motor task (Cordo and Gurfinkel, 2004), reflecting the integrated functional level of the body’s organs. The primary determinants of coordination level include the coupling and coordination of excitatory and inhibitory transitions within the nervous system, as well as the multi-sensory integration of auditory, visual, vestibular, and proprioceptive inputs (Michel, 2012). Research has shown that when athletes participate in coordination tests, they not only require focused attention, but also rely heavily on cognitive function and perceptual-motor skills (Coutinho et al., 2017). Thus, coordination is also a physical quality that imposes certain demands on cognitive and executive functions.

Compared to the control group. However, for the sport-specific coordination test (SDT), the experimental group exhibited a significant decline relative to the control group (*p* = 0.033). This suggests that, under normal-temperature conditions, MF has a limited effect on general coordination ability, but may pose a certain threat to sport-specific coordination.

The results of the present study showed that the Temperature × MF interaction for coordination performance was significant(F_(1,18)_=18.443,p<0.001,η²p=0.506)indicating that the combined presence of both factors produced greater impairment of coordination performance than either factor alone. Simple effects analysis further clarified the nature of this interaction: under MF conditions, coordination performance in the high-temperature environment was significantly worse than that in the normal-temperature environment (*p* < 0.001,d=1.970; whereas under non-MF conditions, there was no significant difference in coordination performance between the high- and normal-temperature environments (*p* = 0.757. Similarly, under high-temperature conditions, the MF induction group performed significantly worse than the control group in the coordination test(*p* < 0.001,d=1.970);whereas under normal-temperature conditions, there was no significant difference between the MF induction group and the control group (*p* = 0.751). This implies that the impairing effect of MF on coordination is triggered only under high-temperature conditions, rather than producing independent and additive impairments as observed for agility.

Unlike the mechanisms underlying the impairment of agility, coordination exhibited resistance to either MF or high ambient temperature alone, with a significant decline occurring only when both stressors were present simultaneously. Several potential mechanisms may account for this synergistic pattern, though none were directly measured in this study:1)when MF acts alone on the prefrontal cortex and anterior cingulate cortex, the executive functions controlled by these brain regions—including self-regulation, performance monitoring, attention allocation, motor preparation, and decision-making—show declines (Funahashi and Andreau, 2013; Martin et al., 2018; Smith et al., 2018). This suggests that under normal-temperature conditions, MF is merely a potential factor affecting coordination, and its negative effects remain within a controllable range. The central nervous system may still maintain relatively stable coordination performance by reasonably allocating and mobilizing remaining cognitive resources, as the residual cognitive resources in the prefrontal and anterior cingulate cortices may still suffice to meet the cognitive and executive demands of the coordination test, thereby not directly leading to a decline in coordination.2)when high ambient temperature is presented as the sole stressor, it prolongs visual attention reaction time, reduces cerebral blood flow and cerebral oxygenation, decreases oxygen supply, disrupts neurotransmitter levels, and reduces total cognitive resources, leading to a certain degree of cognitive decline (Jiang et al., 2013; Douzi et al., 2020);physiologically, it slows nerve conduction velocity, prolongs the muscle contraction–relaxation cycle, and accelerates lactate accumulation, thereby reducing muscle explosive power. However, the results of this study showed no significant change in coordination performance under these conditions. This suggests that, when exposed to high temperature alone, participants’ cognitive and physiological functions were not catastrophically compromised, and that cognitive resources and physiological adaptive capacity remained sufficient to resist the negative effects of heat and maintain stable coordination performance.3)when MF and high ambient temperature co-exist, MF first consumes the cognitive resource reserves in the prefrontal cortex and anterior cingulate cortex, leaving insufficient capacity to mobilize and reallocate cognitive resources in response to high-temperature-induced cognitive decline. Coupled with reduced cerebral oxygenation and neurotransmitter dysregulation, the combined effects deplete the brain’s available cognitive resources and physiological regulatory capacity, leading to a marked decline in motor control and a sharp deterioration in coordination performance.4)the significantly elevated RPE induced by the combination of high temperature and MF—with the HF condition reaching an RPE of 14.26 ± 0.99 after the coordination test—further consumes athletes’ attentional resources, creating a vicious cycle of “cognitive depletion – heightened perception of effort – impaired motor control,” which serves as an important auxiliary mechanism underlying the synergistic detrimental effect.

In summary, these factors may explain why coordination performance did not change significantly under either stressor alone, but exhibited a combined detrimental effect greater than the sum of individual effects when the two were combined.

### 4.4 Effects of high ambient temperature and mental fatigue on other indicators

In addition to examining the effects of high ambient temperature and MF on athletes’ physical performance, this study also explored potential underlying influencing factors, aiming to provide empirical evidence for the development and expansion of research in the field of MF. Drawing on a large body of previous studies in this area (Smith et al., 2015; Van Cutsem et al., 2017b; Veness et al., 2017; Salam et al., 2018; Staiano et al., 2019; Weerakkody et al., 2021), it has been found that MF does not significantly affect athletes’ pre-exercise motivation levels or physiological indicators such as mean heart rate, peak heart rate, or heart rate variability during exercise. However, changes in exercise performance have been shown to be associated with elevated RPE scores during testing. On this basis, the present study concurrently included VAS-MO and RPE measures to further clarify the possible mechanisms through which MF and high ambient temperature affect agility and coordination. The VAS-MO analysis revealed that the main effect of MF induction was not significant(*F* < 0.01,*p* = 0.958), indicating that MF did not affect athletes’ motivation levels during testing, which is consistent with previous findings.; In contrast, ambient temperature exerted a significant independent negative effect on motivation(*F*_(1,18)_=11.857,*p* = 0.003). Notably, the interaction between high ambient temperature and MF did not reach statistical significance (*p* = 0.072), suggesting that the mechanisms by which the two factors affect motivation are independent of each other, and that MF does not further exacerbate the decline in motivation under high-temperature conditions.

Regarding Ratings of Perceived Exertion (RPE), both high ambient temperature and MF independently elevated RPE scores, with no significant interaction between them. This indicates that high ambient temperature generally increases athletes’ perceived exertion after exercise, while MF independently exacerbates perceived exertion during exercise, suggesting that their negative effects are independent and additive—as evidenced by the HF group showing significantly higher RPE after the coordination test than the other three conditions. This finding provides a new perspective for understanding the mechanisms by which MF affects athletic performance: in competitive practice, even during short-duration technical actions, athletes experience a heightened sense of “effort” due to elevated RPE, which arises not only from cognitive load accumulated before or during competition but also in part from the physiological pressure of high ambient temperature, thereby influencing the execution of subsequent actions.

### 4.5 Limitations and future directions

The present study has several limitations. First, an important limitation of this study is the confounding between ambient temperature and time of day. The normal-temperature experimental sessions were uniformly conducted in the morning (7:30–9:00 AM), while all high-temperature sessions were scheduled in the afternoon (1:00–2:30 PM). As a result, the observed differences between the two temperature conditions cannot be unequivocally attributed to temperature alone; diurnal variations in motor performance, fatigue state, hydration status, food intake, and motivational level may also contribute to the observed effects. This confounding factor should be taken into account when interpreting the findings. In addition, relative humidity also showed clear diurnal differences between morning and afternoon sessions, which may also act as a potential confounding factor alongside ambient temperature.Second, the representativeness of the sample is constrained. Although all participants were female collegiate soccer players with a high competitive level from the same university, their actual competitive level may still differ from that of professional athletes. In addition, male athletes were not included in the study, which warrants caution when generalizing the findings to male soccer populations. Future research should include athletes of different sexes and competitive levels (e.g., professional and amateur) to examine the generalizability of the present findings.Third, menstrual cycle phase and hormonal contraceptive use were not systematically monitored or controlled across participants, which represents a notable limitation of this study. Given that all participants were female athletes, fluctuations in reproductive hormone levels across the menstrual cycle or associated with hormonal contraceptive use may potentially modulate thermoregulatory responses and exercise performance under heat stress. In addition, several other field testing-related confounding factors, including hydration status, food intake, and pre-session sleep quality, were not objectively measured or standardized prior to each testing session, which could have introduced variability in fatigue levels and physical performance outcomes.Fourth, although the 45-minute Stroop task is one of the most representative and commonly used methods for inducing MF in the field of sport science, the cognitive load encountered in actual soccer matches is far more complex, involving multiple dimensions such as tactical decision-making, spatial anticipation, and social interaction (e.g., communication with teammates). Future studies may attempt to develop soccer-specific cognitive tasks with greater ecological validity to more realistically simulate the cognitive demands of match play.Fifth, the in-depth exploration of underlying mechanisms remains insufficient. While the present study confirmed the effects of high ambient temperature and MF on agility and coordination, it did not elucidate the physiological and neural mechanisms behind these effects. Incorporating physiological and neural measures—such as core body temperature, skin blood flow, heart rate variability (HRV), electroencephalography (EEG), or functional near-infrared spectroscopy (fNIRS)—would contribute to a more profound mechanistic understanding of the phenomena observed in this study. Sixth, this study lacked long-term adaptive observations and only revealed the acute effects of a single exposure. Whether the conclusions would change after participants undergo heat acclimatization or cognitive anti-fatigue training requires further longitudinal investigations. Seventh, adopting natural outdoor climatic conditions involves a trade-off between ecological validity and experimental control. While natural settings better approximate real-world soccer training and competition and thus enhance external validity, they inevitably reduce experimental control due to natural fluctuations in temperature, humidity and other parameters that cannot be fully standardized across sessions. Future replication in a controlled climate chamber, with standardized temperature, humidity, airflow and exposure duration, would substantially strengthen internal validity. Eighth, the fixed order of physical tests represents a methodological limitation. The agility test was always completed before the coordination test to standardize procedures, meaning residual fatigue from the first test may have accumulated and influenced coordination performance, potentially more strongly under the combined stress of high temperature and mental fatigue. Future studies should counterbalance test order to eliminate this confounding effect. Ninth, the choice reaction time (CRT) measurement had methodological limitations due to equipment functions. The device’s built-in program automatically excluded erroneous responses and outliers, but no explicit quantitative exclusion criteria were specified in the product manual. Trial-level raw data, the number of excluded trials, and accuracy rates were not recorded, so we cannot fully rule out a potential speed-accuracy trade-off in the CRT results. Tenth, the fixed order of physical tests represents a methodological limitation. The agility test was always completed before the coordination test to standardize procedures, meaning residual fatigue from the preceding test may have accumulated and influenced subsequent coordination performance, and this effect may have been more prominent under the combined stress of high ambient temperature and mental fatigue. Future studies should counterbalance the order of the two tests to eliminate this confounding effect and more precisely verify the interaction effect.Finally, although the use of natural summer climatic conditions for high-temperature interventions enhanced the external validity of the study, factors such as ambient temperature, humidity, and sunlight were subject to uncontrollable diurnal fluctuations. Precise control of all environmental parameters in an artificial climate chamber would be an option for achieving higher internal validity in future research.

## 5 Conclusions

The effects of high ambient temperature and MF on agility and coordination in female soccer players differ in their patterns. Both factors exert independent and additive detrimental effects on agility; for coordination, however, a significant interaction effect was observed, whereby a single factor alone is insufficient to impair coordination performance, but the coexistence of both factors leads to a marked decline in coordination. On this basis, the present study enriches the theoretical framework of MF affecting athletic performance under high ambient temperature, and provides scientific guidance for pre-match cognitive load management and training arrangements in summer soccer events.

The findings suggest that, during the pre-competition and in-competition periods of hot-weather tournaments and training sessions, not only are necessary cooling measures required, but also athletes’ pre-match cognitive load should be carefully managed. In particular, prior to technical actions that place high demands on agility and coordination—such as passing and ball control—prolonged exposure to sustained high cognitive-load activities comparable to the 45-min Stroop task (e.g., extended tactical video analysis, intensive pre-match cognitive drills) should be minimized to preserve motor performance and ensure that athletes maintain an optimal competitive state.

## Data Availability

The original contributions presented in the study are included in the article/supplementary material. Further inquiries can be directed to the corresponding author.

## References

[B5] Adams W. M. Hosokawa Y. Casa D. J. (2016). Body-cooling paradigm in sport: maximizing safety and performance during competition. J. Sport Rehabil. 25, 382–394. doi: 10.1123/jsr.2015-0008 27632890

[B25] Amiri-Khorasani M. Sahebozamani M. Tabrizi K. G. (2010). Acute effect of different stretching methods on illinois agility test in soccer players. J. Strength Conditioning Res. 24, 2698–2704. doi: 10.1519/jsc.0b013e3181bf049c 20168255

[B26] Aouadi R. Mehdi R. Khlifa R. (2011). Relationship between Illinois agility test and speed with changes of directions in adult soccer athletes. Fundam. Clin. Pharmacol. 25, 22–23.

[B63] Atha W. F. (2013). Heat-related illness. Emergency Med. Clinics North. America 31, 1097–109+. doi: 10.1016/j.emc.2013.07.012 24176481

[B20] Badin O. O. Smith M. R. Conte D. (2016). Mental fatigue: impairment of technical performance in small-sided soccer games. Int. J. Sports Physiol. Perform. 11, 1100–1105. doi: 10.1123/ijspp.2015-0710 27003948

[B24] Bajaj J. S. Thacker L. R. Heuman D. M. (2013). The Stroop smartphone application is a short and valid method to screen for minimal hepatic encephalopathy. Hepatology 58, 1122–1132. doi: 10.1002/hep.26309 23389962 PMC3657327

[B14] Batista M. M. Paludo A. C. da Silva M. P. (2021). Effect of mental fatigue on performance, perceptual and physiological responses in orienteering athletes. J. Sports Med. Phys. Fitness 61, 673–679. doi: 10.23736/s0022-4707.21.11334-9 33586924

[B44] Bligh J. Johnson K. G. (1973). Glossary of terms for thermal physiology. J. Appl. Physiol. 35, 941–961. doi: 10.1152/jappl.1973.35.6.941 4765838

[B36] Borg G. (2001). Borg’s range model and scales. Int. J. Sport Psychol. 32, 110–126.

[B40] Brietzke C. Franco-Alvarenga P. E. Canestri R. (2020). Carbohydrate mouth rinse mitigates mental fatigue effects on maximal incremental test performance, but not in cortical alterations. Brain Sci. 10 (8), 493. doi: 10.3390/brainsci10080493 32751162 PMC7465505

[B47] Brownsberger J. Edwards A. Crowther R. (2013). Impact of mental fatigue on self-paced exercise. Int. J. Sports Med. 34, 1029–1036. doi: 10.1055/s-0033-1343402 23771830

[B28] Chen Z. Bian C. Liao K. (2021). Validity and reliability of a phone app and stopwatch for the measurement of 505 change of direction performance: A test-retest study design. Front. Physiol. 12. doi: 10.3389/fphys.2021.743800 34630164 PMC8498103

[B59] Cheshire W. P. (2016). Thermoregulatory disorders and illness related to heat and cold stress. Autonomic Neuroscience-Basic Clin. 196, 91–104. doi: 10.1016/j.autneu.2016.01.001 26794588

[B64] Cheung S. S. (2007). Hyperthermia and voluntary exhaustion: Integrating models and future challenges. Appl. Physiol. Nutr. Metab. 32, 808–817. doi: 10.1139/h07-043 17622299

[B56] Coehoorn C. J. Danzy J. Bouchereau-Lal N. (2024). Rapid heat stress causes increased delta spectral power 24 h post-exposure and altered reaction time in firefighters by its dynamic ability to generate heat storage. Fire Saf. J. 146, 104126. doi: 10.1016/j.firesaf.2024.10412642574925

[B65] Cordo P. J. Gurfinkel V. S. (2004). “ Motor coordination can be fully understood only by studying complex movements,” in Brain Mechanisms for the Integration of Posture and Movement: 卷 143. Eds. Mori S. Stuart D. G. Wiesendanger M. ( Elsevier Science Bv, Amsterdam), 29–38. 10.1016/S0079-6123(03)43003-3 14653148

[B67] Coutinho D. Gonçalves B. Travassos B. (2017). Mental fatigue and spatial references impair soccer players’ physical and tactical performances. Front. Psychol. 8. doi: 10.1163/9789004230255_013 28983273 PMC5613114

[B30] Daneshgar-Pironneau S. Audiffren M. Lorcery A. (2025). Endurance athletes are more resistant to mental fatigue than nonathletes. Res. Q. For. Exercise Sport 96, 780–791. doi: 10.1080/02701367.2025.2501972 40455575

[B32] Ding C. Soh K. G. Sun H. (2024). Does mental fatigue affect performance in racket sports? A systematic review. BMC Sports Sci. Med. Rehabil. 16, 179. doi: 10.37766/inplasy2023.2.0017 PMC11345983 39187902

[B6] Douzi W. Dupuy O. Theurot D. (2020). Per-cooling (Using cooling systems during physical exercise) enhances physical and cognitive performances in hot environments. A narrative review. Int. J. Environ. Res. Public Health 17, 1031. doi: 10.3390/ijerph17031031 32041228 PMC7036802

[B3] Draper G. Atkinson G. Chesterton P. (2023). Elite North American soccer performance in thermally challenging environments: An explorative approach to tracking outcomes. J. Sports Sci. 41, 1107–1114. doi: 10.1080/02640414.2023.2259262 37733023

[B60] Fang W. Liu L. Yin B. (2023). Heat exposure intervention, anxiety level, and multi-omic profiles: A randomized crossover study. Environ. Int. 181, 108247. doi: 10.1016/j.envint.2023.108247 37871510

[B50] Fortes L. S. Gantois P. de Lima-Junior D. (2023). Playing videogames or using social media applications on smartphones causes mental fatigue and impairs decision-making performance in amateur boxers. Appl. Neuropsychology-Adult 30, 227–238. doi: 10.1080/23279095.2021.1927036 34061684

[B41] Franco-Alvarenga P. E. Brietzke C. Canestri R. (2019). Caffeine improved cycling trial performance in mentally fatigued cyclists, regardless of alterations in prefrontal cortex activation. Physiol. Behav. 204, 41–48. doi: 10.1016/j.physbeh.2019.02.009 30742838

[B68] Funahashi S. Andreau J. M. (2013). Prefrontal cortex and neural mechanisms of executive function. J. Physiol.-Paris 107, 471–482. doi: 10.1016/j.jphysparis.2013.05.001 23684970

[B13] Gantois P. Ferreira M. E. C. De Lima-Junior D. (2020). Effects of mental fatigue on passing decision-making performance in professional soccer athletes. Eur. J. Sport Sci. 20, 534–543. doi: 10.1080/17461391.2019.1656781 31424354

[B35] Gattoni C. O’Neill B. V. Tarperi C. (2021). The effect of mental fatigue on half-marathon performance: a pragmatic trial. Sport Sci. Health 17, 807–816. doi: 10.21203/rs.3.rs-179643/v142705553

[B31] Hemmat M. Nazemzadegan G. Niknam A. (2025). The effects of mental fatigue and caffeine on basketball performance: insights from a crossover study. J. Int. Soc Sports Nutr. 22, 2541010. doi: 10.1080/15502783.2025.2541010 40726020 PMC12308885

[B42] Ishii A. Tanaka M. Watanabe Y. (2014). Neural mechanisms of mental fatigue. Rev. Neurosci. 25, 469–479. doi: 10.1515/revneuro-2014-0028 24926625

[B52] Jiang Q. Yang X. Liu K. (2013). Hyperthermia impaired human visual short-term memory: An fMRI study. Int. J. Hyperthermia 29, 219–224. doi: 10.3109/02656736.2013.786141 23590362

[B62] Jung W. S. Kim S. W. Park H. Y. (2021). Effects of acute exposure to thermal stress on cardiorespiratory function, skeletal muscle oxygenation, and exercise performance in healthy males. Int. J. Environ. Res. Public Health 18, 7404. doi: 10.3390/ijerph18147404 34299853 PMC8307583

[B4] Kang Z. Chen Z. Liu G. (2024). Can heat conditions affect the heart rate responses, perception of effort, and technical performance of young male football players during small-sided games? a comparative study. BMC Sports Sci. Med. Rehabil. 16, 174. doi: 10.1186/s13102-024-00970-x 39160597 PMC11331650

[B22] Kunrath C. A. Nakamura F. Y. Roca A. (2020). How does mental fatigue affect soccer performance during small-sided games? A cognitive, tactical and physical approach. J. Sports Sci. 38, 1818–1828. doi: 10.1080/02640414.2020.1756681 32362188

[B27] Kutlu M. Yapici H. Yilmaz A. (2017). Reliability and validity of a new test of agility and skill for female amateur soccer players. J. Hum. Kinetics 56, 219–227. doi: 10.1515/hukin-2017-0039 28469760 PMC5384069

[B37] Lakens D. (2013). Calculating and reporting effect sizes to facilitate cumulative science: a practical primer for t-tests and ANOVAs. Front. Psychol. 4. doi: 10.3389/fpsyg.2013.00863 24324449 PMC3840331

[B8] Liu W. H. Li Z. Y. Loh Y. C. (2022). Evaluation of the impact of hot environmental conditions on physical activity among soccer players. Eur. Rev. Med. Pharmacol. Sci. 26, 8216–8223. 10.26355/eurrev_202211_30353 36459005

[B58] Malcolm R. A. Cooper S. Folland J. P. (2018). Passive heat exposure alters perception and executive function. Front. Physiol. 9, 585. doi: 10.3389/fphys.2018.00585 29887804 PMC5981197

[B9] Marcora S. M. Staiano W. Manning V. (2009). Mental fatigue impairs physical performance in humans. J. Appl. Physiol. 106, 857–864. doi: 10.1152/japplphysiol.91324.2008 19131473

[B12] Martin K. Meeusen R. Thompson K. G. (2018). Mental fatigue impairs endurance performance: A physiological explanation. Sports Med. 48, 2041–2051. doi: 10.1007/s40279-018-0946-9 29923147

[B45] Maughan R. J. (2010). Distance running in hot environments: a thermal challenge to the elite runner. Scandinavian J. Med. Sci. Sports 20, 95–102. doi: 10.1111/j.1600-0838.2010.01214.x 21029196

[B16] McKay A. K. A. Stellingwerff T. Smith E. S. (2021). Defining training and performance caliber: A participant classification framework. Int. J. Sports Physiol. Perform. 17, 317–331. doi: 10.1123/ijspp.2021-0451 34965513

[B66] Michel E. (2012). Motor coordination and executive functions. Dev. Med. Child Neurol. 54, 971–971. doi: 10.1111/j.1469-8749.2012.04416.x 22934606

[B43] Migliaccio G. M. Di Filippo G. Russo L. (2022). Effects of mental fatigue on reaction time in sportsmen. Int. J. Environ. Res. Public Health 19, 14360. doi: 10.3390/ijerph192114360 36361239 PMC9656150

[B2] Modric T. Versic S. Stojanovic M. (2023). Factors affecting match running performance in elite soccer: Analysis of UEFA Champions League matches. Biol. Sport 40, 409–416. doi: 10.5114/biolsport.2023.116453 37077779 PMC10108755

[B1] Morgans R. Orme P. Anderson L. (2014). Principles and practices of training for soccer. J. Sport Health Sci. 3, 251–257. doi: 10.1016/j.jshs.2014.07.00242574925

[B48] Morral-Yepes M. Moras G. Bishop C. (2022). Assessing the reliability and validity of agility testing in team sports: A systematic review. J. Strength Conditioning Res. 36, 2035. doi: 10.1519/jsc.0000000000003753 32898034

[B7] Nassis G. P. Girard O. Chiampas G. T. (2024). In-match strategies to mitigate the effect of heat on football (soccer) players’ health and performance. Br. J. Sports Med. 58, 545–56. doi: 10.1136/bjsports-2023-107907 38458625

[B34] Pageaux B. Lepers R. (2018). Chapter 16 - The effects of mental fatigue on sport-related performance Progress in Brain Research: 卷 240 ( Elsevier) (Accessed September 26, 2025). 10.1016/bs.pbr.2018.10.004 30390836

[B54] Pilcher J. J. Nadler E. Busch C. (2002). Effects of hot and cold temperature exposure on performance: A meta-analytic review. Ergonomics 45, 682–698. doi: 10.1080/00140130210158419 12437852

[B51] Pires F. O. Silva-Junior F. L. Brietzke C. (2018). Mental fatigue alters cortical activation and psychological responses, impairing performance in a distance-based cycling trial. Front. Physiol. 9, 227. doi: 10.3389/fphys.2018.00227 29615923 PMC5864900

[B49] Pojskic H. Aslin E. Krolo A. (2018). Importance of reactive agility and change of direction speed in differentiating performance levels in junior soccer players: reliability and validity of newly developed soccer-specific tests. Front. Physiol. 9, 506. doi: 10.3389/fphys.2018.00506 29867552 PMC5962722

[B18] Pryor R. R. Pryor J. L. Vandermark L. W. (2021). Short term heat acclimation reduces heat strain during a first, but not second, consecutive exercise-heat exposure. J. Sci. Med. Sport 24, 768–773. doi: 10.1016/j.jsams.2021.03.020 33846088

[B69] Salam H. Marcora S. M. Hopker J. G. (2018). The effect of mental fatigue on critical power during cycling exercise. Eur. J. Appl. Physiol. 118, 85–92. doi: 10.1007/s00421-017-3747-1 29124324 PMC5754415

[B61] Sawka M. N. Leon L. R. Montain S. J. (2011). Integrated physiological mechanisms of exercise performance, adaptation, and maladaptation to heat stress. Compr. Physiol. 1, 1883–1928. doi: 10.1002/cphy.c100082 23733692

[B15] Smith M. Coutts A. Merlini M. (2015). Mental fatigue impairs soccer-specific physical and technical performance. Med. Sci. sports Exercise 48, 267–276. doi: 10.1249/mss.0000000000000762 26312616

[B23] Smith M. R. Fransen J. Deprez D. (2017). Impact of mental fatigue on speed and accuracy components of soccer-specific skills. Sci. Med. Football 1, 48–52. doi: 10.1080/02640414.2016.125285037339054

[B10] Smith M. R. Thompson C. Marcora S. M. (2018). Mental fatigue and soccer: current knowledge and future directions. Sports Med. 48, 1525–1532. doi: 10.1007/s40279-018-0908-2 29623604

[B38] Smith M. R. Zeuwts L. Lenoir M. (2016). Mental fatigue impairs soccer-specific decision-making skill. J. Sports Sci. 34, 1297–1304. doi: 10.1080/02640414.2016.1156241 26949830

[B46] Smith M. R. Chai R. Nguyen H. T. Marcora S. M. Coutts A. J. (2025). Comparing the effects of three cognitive tasks on indicators of mental fatigue. J. Psychol. 153 (8), 759–83. doi: 10.1080/00223980.2019.1611530 31188721

[B70] Staiano W. Bosio A. Piazza G. (2019). Kayaking performance is altered in mentally fatigued young elite athletes. J. Sports Med. Phys. Fitness 59, 1253–1262. doi: 10.23736/s0022-4707.18.09051-5 30317839

[B11] Thompson C. J. Noon M. Towlson C. (2020). Understanding the presence of mental fatigue in English academy soccer players. J. Sports Sci. 38, 1524–1530. doi: 10.1080/02640414.2020.1746597 32212903

[B21] Trecroci A. Boccolini G. Duca M. (2020). Mental fatigue impairs physical activity, technical and decision-making performance during small-sided games. PloS One 15, e0238461. doi: 10.1371/journal.pone.0238461 32903263 PMC7480836

[B29] Trecroci A. Cavaggioni L. Caccia R. (2015). Jump rope training: balance and motor coordination in preadolescent soccer players. J. Sports Sci. Med. 14, 792–798. PMC4657422 26664276

[B39] Van Cutsem J. De Pauw K. Buyse L. (2017a). Effects of mental fatigue on endurance performance in the heat. Med. Sci. Sports Exercise 49, 1677–1687. doi: 10.1249/mss.0000000000001263 28282326

[B33] Van Cutsem J. Marcora S. De Pauw K. (2017b). The effects of mental fatigue on physical performance: A systematic review. Sports Med. 47, 1569–1588. doi: 10.1007/s40279-016-0672-0 28044281

[B71] Veness D. Patterson S. D. Jeffries O. (2017). The effects of mental fatigue on cricket-relevant performance among elite players. J. Sports Sci. 35, 2461–2467. doi: 10.1080/02640414.2016.1273540 28092208

[B19] Wallenberg N. Lindberg F. Thorsson S. (2023). The effects of warm weather on children’s outdoor heat stress and physical activity in a preschool yard in Gothenburg, Sweden. Int. J. Biometeorol. 67, 1927–1940. doi: 10.1007/s00484-023-02551-y 37726553 PMC10643434

[B57] Ward M. K. Yam C. M. H. Palejwala Z. (2021). An experimental simulation of heat effects on cognition and workload of surgical team members. Ann. Surg. 274, E395–E402. doi: 10.1097/sla.0000000000004598 33196490

[B53] Weerakkody N. S. Taylor C. J. Bulmer C. L. (2021). The effect of mental fatigue on the performance of Australian football specific skills amongst amateur athletes. J. Sci. Med. Sport 24, 592–596. doi: 10.1016/j.jsams.2020.12.003 33386238

[B17] Yang H. Ren J. Chen X. (2026). Study of outdoor exercise in high temperature environments and recovery time recommendations based on physiological strain index. Int. J. Biometeorol. 70, 5. doi: 10.1007/s00484-025-03104-1 41483221

[B55] Yeganeh A. J. Reichard G. McCoy A. P. (2018). Correlation of ambient air temperature and cognitive performance: A systematic review and meta-analysis. Build. Environ. 143, 701–716. doi: 10.1016/j.buildenv.2018.07.00242574925

